# Physiological and Transcriptomic Analysis Reveals the Responses and Difference to High Temperature and Humidity Stress in Two Melon Genotypes

**DOI:** 10.3390/ijms23020734

**Published:** 2022-01-10

**Authors:** Jinyang Weng, Asad Rehman, Pengli Li, Liying Chang, Yidong Zhang, Qingliang Niu

**Affiliations:** School of Agriculture and Biology, Shanghai Jiao Tong University, Shanghai 200240, China; wengjinyang@sjtu.edu.cn (J.W.); asadrehman@sjtu.edu.cn (A.R.); lipengli@sjtu.edu.cn (P.L.); changly@sjtu.edu.cn (L.C.); zhyd@sjtu.edu.cn (Y.Z.)

**Keywords:** melon, high temperature and humidity stress, transcriptome, chlorophyll fluorescence parameters, chloroplast ultrastructure

## Abstract

Due to the frequent occurrence of continuous high temperatures and heavy rain in summer, extremely high-temperature and high-humidity environments occur, which seriously harms crop growth. High temperature and humidity (HTH) stress have become the main environmental factors of combined stress in summer. The responses of morphological indexes, physiological and biochemical indexes, gas exchange parameters, and chlorophyll fluorescence parameters were measured and combined with chloroplast ultrastructure and transcriptome sequencing to analyze the reasons for the difference in tolerance to HTH stress in HTH-sensitive ‘JIN TAI LANG’ and HTH-tolerant ‘JIN DI’ varieties. The results showed that with the extension of stress time, the superoxide dismutase (SOD), peroxidase (POD), and ascorbate peroxidase (APX) activities of the two melon varieties increased rapidly, the leaf water content increased, and the tolerant varieties showed stronger antioxidant capacity. Among the sensitive cultivars, Pn, Fv/Fm, photosystem II, and photosystem I chlorophyll fluorescence parameters were severely inhibited and decreased rapidly with the extension of stress time, while the HTH-tolerant cultivars slightly decreased. The cell membrane and chloroplast damage in sensitive cultivars were more severe, and Lhca1, Lhca3, and Lhca4 proteins in photosystem II and Lhcb1-Lhcb6 proteins in photosystem I were inhibited compared with those in the tolerant cultivar. These conclusions may be the main reason for the different tolerances of the two cultivars. These findings will provide new insights into the response of other crops to HTH stress and also provide a basis for future research on the mechanism of HTH resistance in melon.

## 1. Introduction

In recent years, with the increase in the global greenhouse effect and climate change, the global temperature fluctuations have intensified, and the frequency of extremely high temperatures and heavy rain has increased significantly in summer [1,2]. In China, the arrival of typhoons often causes continuous rainfall for several days in summer, which leads to increases in air humidity and soil humidity. Coupled with extremely high-temperature weather, the environment of high temperature and high humidity has a serious impact on the growth and quality of plants [3]. At present, one of the biggest challenges facing sustainable agriculture is to cultivate and modify the potential characteristics of adaptive crops. Different crop varieties have different stress resistances, which provides relevant evidence for genetic resources [4]. Therefore, it is of great significance to study the physiological changes and molecular mechanisms of vegetable crops in the resistance to extremely high-temperature and high-humidity environments to improve the tolerance of vegetables and breed new varieties.

Melon (*Cucumis melo* L.) is one of the most popular fruits because of its high economic and nutritional value. Melon is usually planted in two crops a year in China. Melon seedlings often suffer from high temperature and high humidity in summer, and a large number of melons die due to the influence of short and extremely high temperatures and high humidity, causing huge economic losses. In recent decades, the effects of heat stress on plants have been widely studied, but few studies have investigated the combination of high temperature and high-humidity stress. Previous studies on the changes in lettuce under high-temperature stress found that high temperature inhibited the growth of lettuce seedlings, resulting in reduced chlorophyll content and net photosynthesis, which destroyed chloroplast and mitochondrial structures [5]. Heat stress reduces the photosynthetic capacity of leaves and thus destroys the photosystem II within the photosynthetic apparatus [6]. Heat stress often causes oxidative stress, which leads to the accumulation of reactive oxygen species (ROS) and serious damage to plant organelles [7]. To maintain the growth of plants under high temperature, the antioxidant system will be activated quickly, and the activity of enzymes involved in scavenging the ROS system will increase to effectively remove ROS, prevent membrane lipid peroxidation, and maintain the balance of ROS in plants [8].

Chlorophyll fluorescence parameters are often used to identify genotypes with different stress tolerance and to study the photosynthetic response mechanism under different stress conditions [9,10]. Previous studies on potential heat-resistant chickpea genotypes using chlorophyll fluorescence parameters found that heat-resistant chickpea showed higher Fv/Fm, Fq’/Fm’, Pn, and seed yield at warm sites [11]. One study showed that Fm, Fv/Fm, ΦPSII, Chl, and qP decreased, while NPQ, minimum fluorescence (Fo), and anthocyanin (Anth) content increased under high light intensity and severe water stress in *Aloe vera* L. [12]. It was found that the electron flow of photosystem II (PSII) was decreased in two orchids after fluctuating light treatment under heat stress, and the effects of heat stress on plant photosynthetic systems were species-dependent [13]. Studies have shown that plants under stress can change the distribution of light energy absorbed by leaves, and it is of great significance to analyze the changes and effects of photosystem II and photosystem I under stress by detecting related parameters of chlorophyll fluorescence, which is of great significance to study the regulatory mechanism of the response of the photosynthetic apparatus [14].

When plants are faced with multiple stresses at the same time, they develop complex defense mechanisms to recognize and respond to them [15]. Melon also has adaptation mechanisms to environmental stress during long-term natural domestication, different varieties have different adaptive mechanisms to high temperature and high-humidity stress, and plant responses to high-temperature and high-humidity stress are species-dependent. Therefore, it is very important to study the response of different varieties to HTH stress [16]. In addition to physiological responses, some molecular regulatory pathways and important regulatory genes are involved in response to high temperature and humidity stress. Previous studies have been shown that carbohydrate transport and metabolism, photosynthesis, signal transduction mechanisms, pathogen resistance, and secondary metabolites were involved in defense mechanisms that improve the heat resistance of the plant [17,18]. The *CaZNF830* acts as a positive regulator of tolerance to high temperature and high humidity (HTHH) and activates many defense-linked genes, improving the tolerance to HTHH in pepper [19]. It has been reported that the overexpression of the *Gm1-MMP* of bean in *Arabidopsis* can enhance leaf and developing seed tolerance, as well as tolerate the HTH stress [20]. At present, there are few studies on the mechanism of HTH tolerance in plants. Therefore, studying the physiological response of different melon varieties under HTH stress is the basis for revealing the mechanism of resistance to HTH stress.

In this study, we selected varieties with HTH-sensitive ‘JIN TAI LANG’ and HTH-tolerant ‘JIN DI’ by comparing and measuring the changes in physiological indexes, photosynthetic indexes, and chlorophyll fluorescence parameters under different stress treatments, by further analysis of two different genotype varieties of HTH stress in response to physiological differences and combined with RNA-seq analysis the DEGs of two varieties under stress, revealed two different genotypes of melon with differences in the HTH stress response mechanism. Thus, it is of great significance to elucidate the molecular breeding of melon with high temperature and humidity tolerance in the future.

## 2. Material and Methods

### 2.1. Plant Materials and HTH Treatments

On the basis of our previous study, the HTH-sensitive ‘JIN TAI LANG’ and HTH-tolerant ‘JIN DI’ varieties were used as plant materials to study the response to HTH stress [21]. Melons seeds with the same shape and size were selected and put into water at 55 °C for high-temperature, sterilized for 30 min, and then placed in a petri dish at 28 °C for germination. When most of the seeds germinated, took the germinated seeds were planted in 21-well seedling trays and placed in the plant growth chamber (RXM Intelligent; Ningbo Jiangnan Instrument Factory, China). The temperature of the growth incubator was set at 30 °C for day and at 20 °C for night (12 h/12 h), the light intensity was set at 600 μmol m^−2^ s^−1,^ and air humidity was set at 60%. When the melon seedlings grew to three true leaves stage, the melon seedlings were treated with HTH stress. The temperature was set at 45 °C and at 35 °C (12 h/12 h) for day and night, respectively. Soil moisture was set at 100%, the light intensity was adjusted at 600 μmol m^−2^ s^−1^, and air humidity was set at 85% for day and 95% for night [22]. The second true leaf of the two cultivars was collected at 0, 1, 2, 3, and 4 days of treatment for physiological and biochemical indexes and RNA-seq analysis, and each index was repeated at least three times.

### 2.2. Measurements of Agronomic Traits

Plant height (PH), leaf length (LL), leaf width (LW), and stem diameter (SD) of the two varieties were measured under different HTH stress days. PH was the height from the soil surface to the flag leaf; LL and LW were determined as the distance of the third leaf from tip to petiole and the width of the middle, respectively; SD was determined as the width of the stem below the cotyledons by a Vernier caliper.

### 2.3. Determination of Physiological and Biochemical Indexes

The superoxide dismutase (SOD) activity, catalase (CAT) activity, peroxidase (POD) activity, ascorbate peroxidase (APX), superoxide anion (O^2−^) content, and H_2_O_2_ content were determined by using assay kits (Comin, Suzhou, China) [23,24]. The method of determination of total chlorophyll content was described by Cao et al. [25]. The method for determining the relative conductance (REC) was described by Aghaie et al. [26]. Malondialdehyde (MDA) content was measured according to Shah et al. [27]. Soluble protein content was measured using coomassie brilliant blue G-250 and the binding protein chromogenic method, the method defined by Bradford [28]. Leaf water content (LWC) was calculated as LWC = (WF − WD)/WF × 100%. The dry weights (WD) and fresh (WF) were determined using an electronic analytical balance. Dry weight is the weight of the leaves placed in the oven at 80 °C until they dry [29].

### 2.4. Gas Exchange Parameters, Chlorophyll Fluorescence, and P700 Measurements

The main gas exchange parameter, net photosynthetic rate (Pn), transpiration rate (E), stomatal conductance (Gs), and intercellular carbon dioxide concentration (Ci) were selected and determined by using the GFS-3000 System (WALZ, Effeltrich, Germany). The main photosynthesis system parameters were set as follows: the leaf chamber temperature and humidity were set at 25 °C and 60%, respectively. Light intensity was set at 600 μmol m^−2^ s^−1^, and the air velocity was set at 750 μmol s^−1^. The change value of CO_2_ concentration was between 420 and 460 ppm during measurement. The second leaf of melon seedling was used for parameter determination.

To determine the chlorophyll fluorescence parameters of photosystems PSII and PSI at 25 °C by the Dual-PAM-100 fluorescence system (WALZ, Effeltrich, Germany). The second true leaf of melon seedling was selected for measurement, and the measurement was carried out after 30 min of dark adaptation. A saturating pulse (20,000 μmol photons m^−2^ s^−1^, 300 ms) was measured the maximum fluorescence and the maximum change in P700, and then an actinic light of 358 μmol photons m^−2^ s^−1^ was used to illuminate the leaves for 5 min to activate the electron sink in photosynthesis, saturation pulses were generated every 20 s. The Fv/Fm, qP, NPQ, Y(II), and ETR(II) of PSII parameters were calculated according to Kramer et al. [30] and Su et al. [31]; the Y(I), ETR(I), Y(ND) and Y(NA) of PSI parameters were calculated according to Schreiber and Klughammer. [32] and Tan et al. [33].

### 2.5. Chloroplast Ultrastructure by Transmission Electron Microscopy (TEM)

The fresh leaves of the second true leaf of sensitive and tolerant cultivars on the third day of stress were collected and observed by TEM. The sample fixation and sectioning, and staining methods were determined according to Cheng et al. [34]. The ultrastructure and morphology of chloroplasts were observed at microscope scales of 5, 2, and 1 μm by using a transmission electron microscope (Tecnai G2 SpiritBiotwin, FEI).

### 2.6. RNA Extraction and Sequencing

The second true leaf of HTH-sensitive ‘JIN TAI LANG’ and HTH-tolerant ‘JIN DI’ varieties on the third day of stress was selected to be used for RNA-seq analysis. Total RNA was extracted from plant leaves according to the operation method of RNAprep Pure Plant Plus Kit (Tiangen, Beijing, China). Then, high-quality RNA samples that have been tested were constructed RNA-seq libraries and by using the Illumina NovaSeq 6000 (2 × 150 bp read length) for transcriptome sequencing (Majorbio, Shanghai, China). Quality control was carried out on the raw sequencing data by Sickle (https://github.com/najoshi/sickle, accessed on 10 January 2021) and SeqPrep (https://github.com/jstjohn/SeqPrep, accessed on 10 January 2021), then the clean reads were mapped to Cucumis_melo reference genome (http://cucurbitgenomics.org/organism/18, accessed on 10 January 2021) by HISAT2 software [35] and assembled [36].

The DESeq2 with Q value ≤ 0.05, DEGs with |log2FC| ≥ 1 and *p*-value < 0.05 were used for group differential gene analysis [37] and use FPKM (expected number of fragments per kilobase of transcript sequence per million base pairs sequenced) to compare different expression genes (DEGs) in two samples [38]. In addition, GO functional and KEGG pathway enrichment analyses were according to Xie et al. [39]. A total of six cDNA libraries were constructed and sequenced with three biological replicates for each sample. The RNA sequence data set are available in the repository of NCBI Sequence Read Archive (SRA) under the GenBank accession BioProject: PRJNA775054 and accession number: SRR16596901-16596906.

### 2.7. qRT-PCR Analysis

To evaluate the accuracy and repeatability of the sequencing data, 15 genes with significant differences were selected for qRT-PCR validation. The total RNA was extracted from different tissues by using RNAprep Pure Plant Plus Kit (Tiangen, Beijing, China). The first-strand cDNA was synthesized with 1 μg RNA by using FastKing RT Kit (With gDNase) (Tiangen, Beijing, China). The Talent qPCR PreMix (SYBR Green) (Tiangen, Beijing, China) was used for qRT-PCR by a Bio-Rad CFX Connect Real-Time System machine (Bio-Rad, Hercules, CA, USA). The PCR condition was set as follows: 3 min at 95 °C for initial denaturation, and 40 cycles of PCR (95 °C for 5 s, 60 °C for 10 s, and 72 °C for 15 s). Each sample was represented by three biological replicates and three technical replicates. The relative expression was calculated using the 2^−ΔΔCt^ method [40]. Actin was used as an internal control (reference gene). The primers used for qRT-PCR are listed in Supplementary Table S1.

### 2.8. Statistical Analysis

The effects of different days of stress on the physiological and photosynthetic parameters of melon seedlings under HTH stress were analyzed by one-way ANOVA. Significance was calculated by Duncan’s multiple range test at the *p* < 0.05 level in SPSS 25.0 (IBM, Chicago, IL, USA). All data were analyzed by Microsoft Excel 2016, and figures were made by GraphPad Prism software (version 8.0.0 for Windows, San Diego, CA, USA).

## 3. Results

### 3.1. Changes in Growth Morphological Indices of the Two Melon Varieties

**Figure 1:**
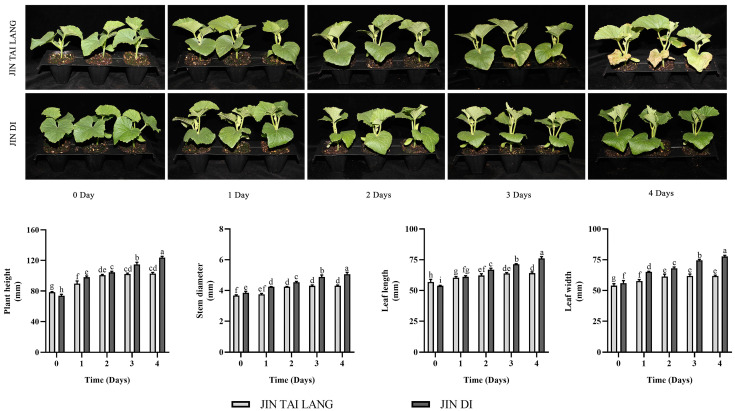
Changes in the melon growth morphology index on different days under HTH stress. Different letters indicate significant differences according to Duncan’s multiple range test at *p* < 0.05 using a one-way ANOVA. Values are represented as the means ± SD (*n* = 6).

Plants usually produce a series of physiological responses to maintain normal growth under stress. As shown in Figure 1, the growth morphological indexes of the two varieties of melon showed different forms under HTH stress on different days. The HTH-sensitive variety ‘JIN TAI LANG’ grew rapidly on the first and second days of stress. From the third day of stress, the first leaf began to turn yellow under HTH stress, and on the fourth day of stress, the first leaf turned yellow completely, the second leaf was severely stressed, and the whole plant stopped growing and gradually died. On the other hand, in the ‘JIN DI’ variety, which is resistant to HTH stress, from the first day to the fourth day of HTH stress, the melon seedlings continued to grow, and the leaf stress phenotype was not obvious, showing suitable resistance to HTH stress.

### 3.2. Changes in Antioxidant Enzymes in Two Different Genotypes of Melon Cultivars under HTH Stress

**Figure 2:**
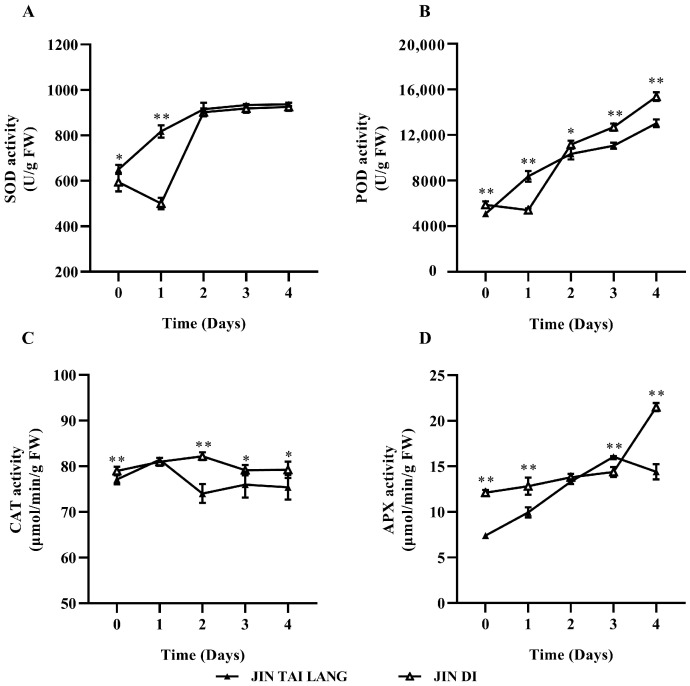
Effects of HTH treatment on antioxidant enzyme activities in different melon seedlings. (**A**), superoxide dismutase (SOD) activity; (**B**), peroxidase (POD) activity; (**C**), catalase (CAT) activity; (**D**), ascorbate peroxidase (APX) activity. Significant different levels of antioxidant enzymes in melon seedlings under different days of stress: * (*p* < 0.05), ** (*p* < 0.01). Values are represented as the means ± SD (*n* = 4).

The SOD activity of seeding leaves of the two melon varieties seedings leaf increased rapidly from 0 to 2 days under HTH stress treatment and increased slowly from 2 to 4 days. The enzyme activity of both varieties reached the highest level after 4 days of stress (Figure 2A). The POD activity increased rapidly in both cultivars, the POD activity increased faster in the HTH-tolerant cultivar ‘JIN DI’ than that in the HTH-sensitive cultivar ‘JIN TAI LANG’, and the POD activity was significantly higher than that in the HTH-sensitive cultivar at 4 days of stress (Figure 2B). The change in CAT activity of the ‘JIN TAI LANG’ cultivar showed a trend of “M”, a general downwards trend (Figure 2C). The CAT activity of the HTH-tolerant cultivar ‘JIN DI’ first increased and then decreased, and it was significantly higher than that of the HTH-sensitive cultivar at the later stage of HTH stress (Figure 2C). The APX activity increased rapidly from 0 to 3 days in the two melon varieties, but the HTH-sensitive cultivar decreased rapidly on the fourth day of stress. On the other hand, the APX activity in ‘JIN DI’ seedings increased slowly at the beginning and rapidly at the later stage of stress and was significantly higher than that of the sensitive variety (Figure 2D). In conclusion, the SOD, POD, and APX activities of the two melon varieties on day 4 of stress were higher than those of the seedlings without treatment under HTH stress. The results showed that the two melon varieties were resistant to HTH stress by increasing antioxidant enzyme activity to remove excess reactive oxygen species and anions. The POD, CAT, and APX activities of the HTH-tolerant cultivar were significantly higher than those of the sensitive cultivar.

### 3.3. Changes of Physiological Indices of Two Different Genotypes of Melon Varieties Changed under HTH Stress

**Figure 3:**
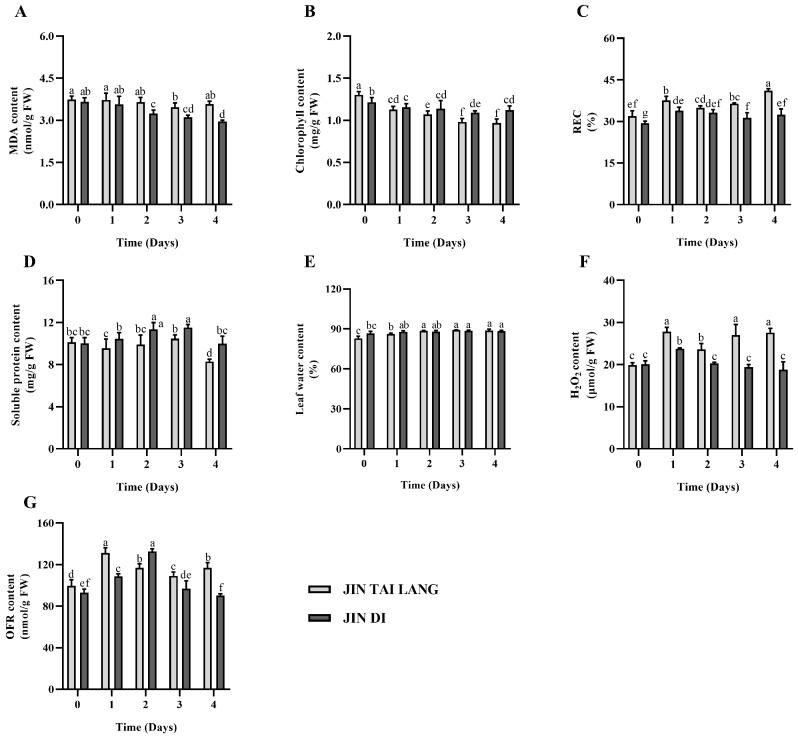
Physiological indexes of two different genotypes of muskmelon under HTH stress. (**A**), malondialdehyde (MDA) content; (**B**), chlorophyll content; (**C**), relative conductance (REC) (%); (**D**), soluble protein content; (**E**), leaf water content (%); (**F**), hydrogen peroxide (H_2_O_2_) content; (**G**), superoxide anion (OFR) content. Different letters indicate significant differences according to Duncan’s multiple range test at *p* < 0.05 using a one-way ANOVA. Values are represented as the means ± SD (*n* = 4).

MDA is a product of membrane lipid peroxidation, and its content reflects the degree of membrane lipid peroxidation, which is closely related to the degree of damage caused by crop stress or senescence. Under the stress of high temperature and humidity, the ‘JIN TAI LANG’ variety with MDA content reduced at the beginning and then increased. In both varieties, leaf MDA content after 4 days of stress was lower than those in untreated melon seedling leaves (Figure 3A). The chlorophyll content in both melon cultivars showed a downward trend, and the chlorophyll content in the ‘JIN TAI LANG’ variety was significantly lower than that in the ‘JIN DI’ variety (Figure 3B). The relative electrical conductivity of the two melon varieties increased significantly with different days of HTH treatment. At 4 days of stress, the relative conductivity of the two cultivars was significantly higher than that of the untreated seedlings, and the relative conductance of the ‘JIN TAI LANG’ variety was significantly higher than that of the ‘JIN DI’ variety, indicating that the cell membranes of both cultivars were damaged, and those of the ‘JIN TAI LANG’ variety were seriously damaged (Figure 3C). The content of soluble protein in the leaves of both melon varieties initially increased and then decreased. On the fourth day of stress, the content of soluble protein in leaves of ‘JIN TAI LANG’ variety was significantly lower than that on day 0 and the ‘JIN DI’ variety, and there was no significant difference in soluble protein content between ‘JIN DI’ variety and 0-day soluble protein content (Figure 3D). Under HTH stress, the leaf water content of the two cultivars increased significantly, and the increase in leaf water content of the ‘JIN TAI LANG’ variety was significantly higher than that of the ‘JIN DI’ variety (Figure 3E). Under different days of HTH treatment, the H_2_O_2_ content of the ’JIN TAI LANG’ variety increased continuously, while the H_2_O_2_ content of HTH-tolerant varieties showed a trend of first increasing and then decreasing (Figure 3F). The content of OFR in both cultivars first increased and then decreased. There was no significant difference between the ‘JIN DI’ variety and the untreated seedlings on the fourth day of stress, while the content of OFR in the ‘JIN TAI LANG’ variety increased on the fourth day to a level that was significantly higher than that in the ‘JIN DI’ variety (Figure 3G).

### 3.4. Effects of HTH Stress on Photosynthetic Gas Exchange Parameters of Two Different Melon Genotypes

**Figure 4:**
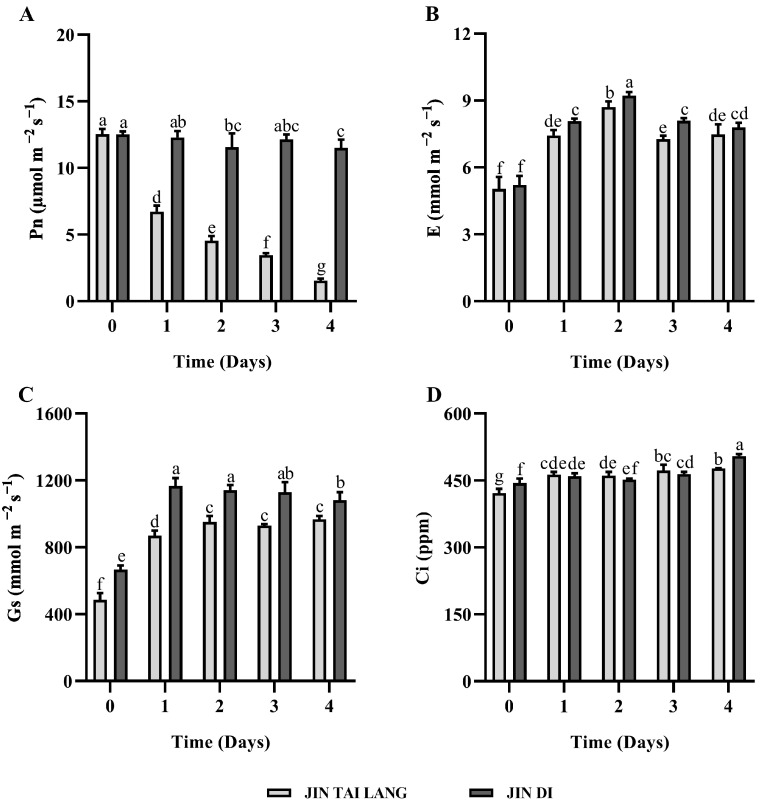
Changes of gas exchange parameters in leaves of two melon seedlings under HTH stress. (**A**), net photosynthetic rate(Pn); (**B**), transpiration rate (E); (**C**), stomatal conductance (Gs); (**D**), intracellular CO2 concentration (Ci). Different letter indicates significant differences according to Duncan’s multiple range test at *p* < 0.05 using a one-way ANOVA. Values are represented as the means ± SD (*n* = 6).

Under HTH stress, the Pn of the ‘JIN TAI LANG’ variety decreased rapidly with the increasing stress time, which significantly inhibited the plants (Figure 4A). The Pn of the ‘JIN DI’ variety decreased slightly under stress and maintained a higher photosynthetic rate(Figure 4A). Transpiration rate (E) increased first and then decreased, and E increased under high temperature and humidity treatment (Figure 4B). The Gs increased rapidly in both cultivars and remained high, increasing gas exchange to resist stress (Figure 4C). The Ci of the two cultivars increased significantly under stress, and that of the ‘JIN TAI LANG’ variety was significantly lower than that of the ‘JIN DI’ variety (Figure 4D).

### 3.5. Effects of HTH Stress on Fluorescence Parameters of Two Different Melon Genotypes

**Figure 5:**
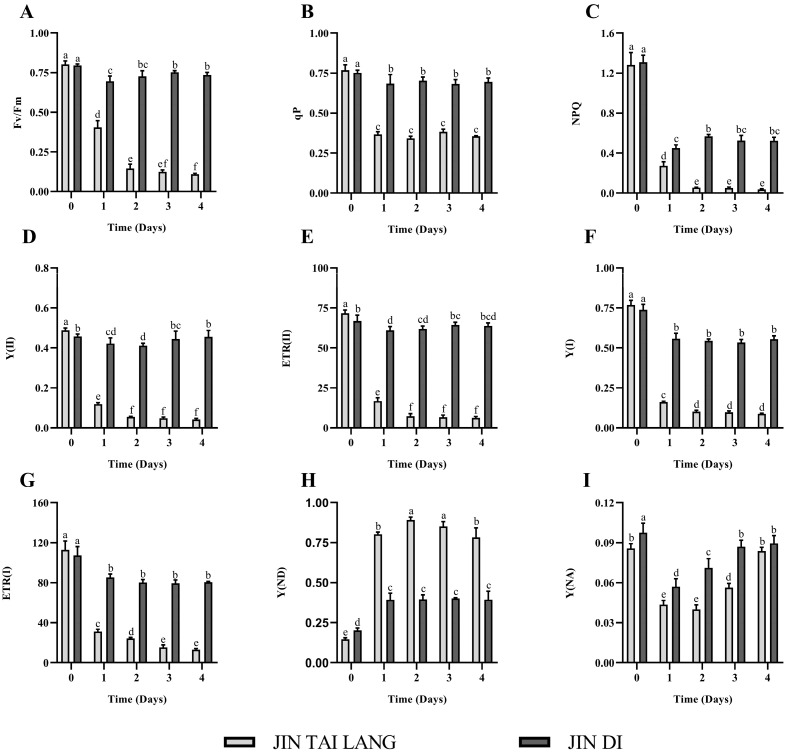
Changes in the leaf fluorescence parameters of two melon genotypes seedlings under HTH stress. (**A**), the maximum quantum yield of PSII (Fv/Fm); (**B**), photochemical quenching (qP); (**C**), non-photochemical quenching (NPQ); (**D**), the effective quantum yield of PSII (Y(II)); (**E**), the electron transport rate through PSII (ETRII); (**F**), the quantum yield of PSI photochemistry (Y(I)); (**G**), the electron transport rate through PSI (ETRI); (**H**), the quantum yield of PSI non-photochemical energy dissipation due to donor-side limitation, (Y(ND)); (**I**), the quantum yield of non-photochemical energy dissipation due to acceptor side limitation (Y(NA)). Different letter indicates significant differences according to Duncan’s multiple range test at *p* < 0.05 using a one-way ANOVA. Values are represented as the means ± SD (*n* = 6).

The PSII fluorescence parameters are one of the important indices to reflect the degree of plant stress. Under HTH stress treatment, the Fv/Fm values of the two melon varieties were not consistent. With the extension of stress time, the value of Fv/Fm of the ‘JIN TAI LANG’ variety decreased rapidly, while the value of Fv/Fm of the‘JIN DI’ variety decreased first and then slightly increased, maintaining a suitable state (Figure 5A). The qP is the photochemical quenching coefficient, which reflects the openness of the PSII reaction center. The qP of both melon varieties decreased significantly under stress, while the qP value of the ‘JIN DI’ variety was significantly higher than that of the sensitive variety, maintaining a high photochemical quenching for photosynthesis (Figure 5B). NPQ is the excitation energy absorbed by photosystem II and dissipates the heat energy through a regulatory photoprotection mechanism. Under HTH stress, the NPQ of both cultivars decreased rapidly and significantly, while the NPQ of the ‘JIN TAI LANG’ variety was lower, indicating that the photoprotection mechanism was seriously damaged under stress (Figure 5C). Y(II) and ETR(II) represent the actual photosynthetic efficiency of photosystem II and the relative electron transfer rate of photosystem II, respectively. Under high-temperature stress, the Y(II) and ETR(II) of the ‘JIN TAI LANG’ variety decreased rapidly, and the values were low after 4 days of stress, and the photosynthetic efficiency and relative electron transfer were severely inhibited. The Y(II) and ETR(II) of the ‘JIN DI’ variety decreased significantly under stress and maintained a high level to maintain the normal photosynthesis of plants (Figure 5D,E).

The photosystem I indices, Y(I) and ETR(I) of the two melon varieties were significantly decreased, indicating that the actual photosynthetic efficiency and relative electron transfer for photosystem I were significantly inhibited and that photosystem I was damaged (Figure 5F,G). Y(ND) is the quantum yield of non-photochemical energy dissipation caused by donor-side restriction of photosystem I. Under stress, the Y(ND) of the two cultivars increased, and that of the‘JIN TAI LANG’ variety was significantly higher than that of the ‘JIN DI’ variety (Figure 5H). Y(NA) is the quantum yield of non-photochemical energy dissipation caused by receptor side restriction of photosystem I. Under HTH stress, Y(NA) decreased first and then increased in the two melon varieties, indicating that photosystem I was subjected to increasingly severe stress with time (Figure 5I).

### 3.6. Effects of HTH Stress on Chloroplast Ultrastructure

**Figure 6:**
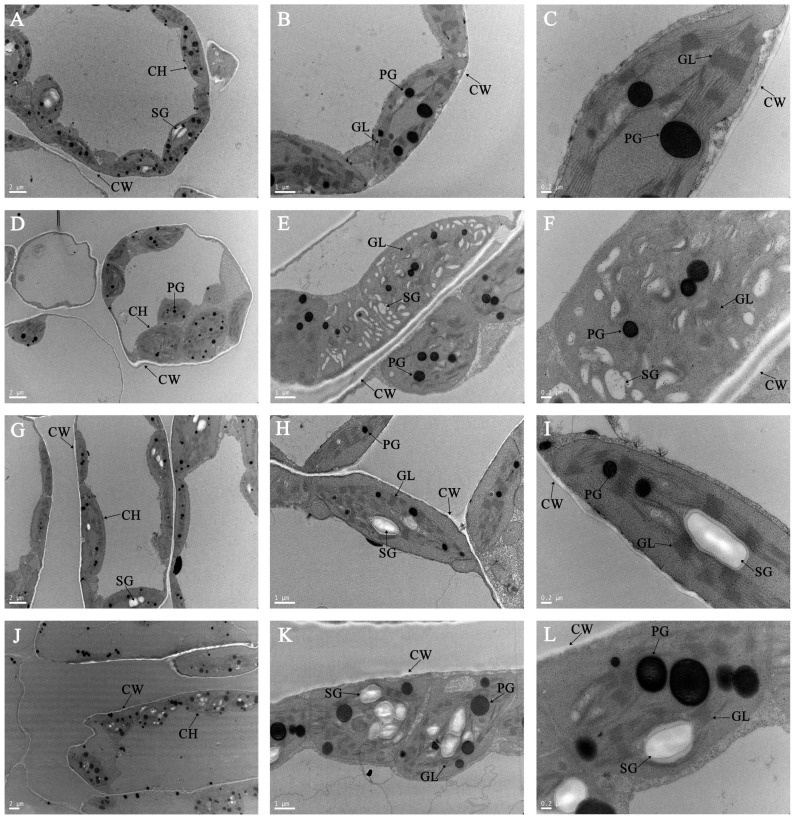
Transmission electron micrographs (TEM) of chloroplasts at low power microscope scales of 2 μm, 1 μm, or 0.2 nm at 3 days of HTH stress treatments and untreated seedlings (0 day) in melon seedings leaves. (**A**–**F**) represent the chloroplast ultrastructure of the ‘JIN TAI LANG’ variety under HTH stress at 3 days and untreated seedling (0 day), respectively; (**G**–**L**) represent the chloroplast ultrastructure of the ‘JIN DI’ variety under HTH stress at 3 days and untreated seedling (0 day), respectively. CH, chloroplast; CW, cell wall; SG, starch granule; GL, grana lamellae; PG, plastoglobule. Bars indicate 2 μm, 1 μm, and 0.2 nm.

The leaf cells of two healthy melon varieties under natural conditions contained a large number of chloroplasts lining the inner walls of the cells (Figure 6A,G). Chloroplasts are long ellipsoid or irregularly spherical, and there are a few starch grains and a few plastoglobules in chloroplasts. The grana and stromal thylakoid were arranged neatly, and the lamellar structure was compact and intact (Figure 6B,C,H,I). At three days of HTH stress, the ultrastructure of chloroplasts in the leaves of the ‘JIN TAI LANG’ variety was severely damaged, which was mainly manifested in the disappearance of a large number of chloroplasts in the cells, cell deformation, deformation of chloroplast structure, expansion of volume, and morphological overbulk (Figure 6D). A large number of starch particles appear in chloroplasts (Figure 6E), loose arrangement of matrix lamellae, serious loose grana lamellae, and degradation (Figure 6F). In the ‘JIN DI’ variety, the chloroplasts in cells decreased slightly, and the chloroplasts showed slight enlargement and deformation (Figure 6J), the number of starch grains and plastoglobules increased (Figure 6K), and the stroma and grana lamella were slightly loose (Figure 6L). In general, the cell morphology and chloroplast structure of the ‘JIN TAI LANG’ variety were severely damaged under HTH stress, while those of the ‘JIN DI’ variety were less damaged.

### 3.7. Transcriptome Statistics of DEGs, GO, and KEGG Functional Enrichment Analysis

**Figure 7:**
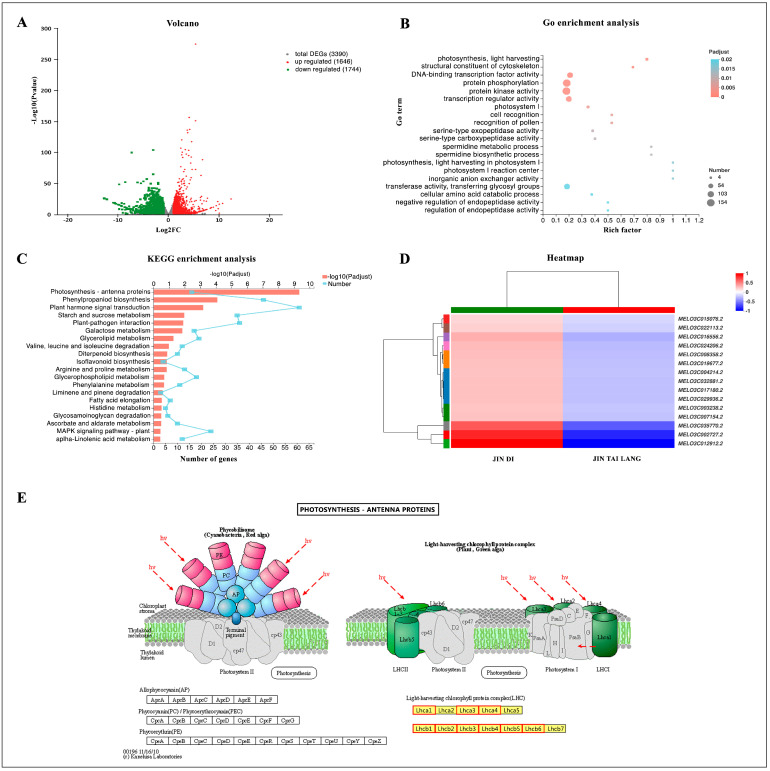
Transcriptome statistics of DEGs, GO, and KEGG functional enrichment analysis of two genotypes of melon varieties during the third day under HTH stress. (**A**), Volcanic map analysis of DEGs of the ‘JIN TAI LANG’ and ‘JINDI’ varieties. (**B**), Top 20 GO enrichment analyses of DEGs in two melon varieties. (**C**), Top 20 KEGG enrichment analyses of DEGs in the two melon varieties. (**D**), Heat map of the 15 most significant DEGs in the KEGG enrichment pathway. The numbers in the scale bar represent the log2 (fold changes) in gene expression. (**E**), Schematic diagram of the most significant KEGG enrichment pathway.

At three days of HTH treatment, the number of differentially expressed genes (DEGs) in leaves of ‘JIN TAI LANG’ and ‘JIN DI’ varieties were counted. Taking the ‘JIN TAI LANG’ variety as a control, a total of 3390 gene expression changes were detected in the two varieties under HTH stress. Among them, there were 1646 up-regulated genes and 1744 down-regulated genes (Figure 7A and Table S2). GO enrichment analysis was performed on DEGs, and the top 20 enrichment categories of DEGs related to HTH stress were identified (Figure 7B). Within those categories, go functions were mainly enriched in “pigment binding”, “light harvesting in photosystem I”, “primary metabolic process”, and “cellular protein modification process”. The KEGG enrichment pathway of DEGs was further analyzed and was mainly enriched in “photosynthesis-antenna proteins”, ”phenylpropanoid biosynthesis”, ”plant hormone signal transduction”, ”starch and sucrose metabolism”, and “plant-pathogen interaction” (Figure 7C), and the function of GO enrichment was similar. By analyzing the KEGG enrichment pathway of the most significant difference, the difference between the two cultivars was mainly in the “light-harvesting protein complex”, in which the Lhca1, Lhca3, Lhca4, and Lhcb1-Lhcb6 in the ‘JIN TAI LANG’ variety were significantly lower than in the ‘JIN DI’ variety (Figure 7E).

### 3.8. Validation of RNA-Seq Results by Quantitative Real-Time PCR (qRT-PCR)

**Figure 8:**
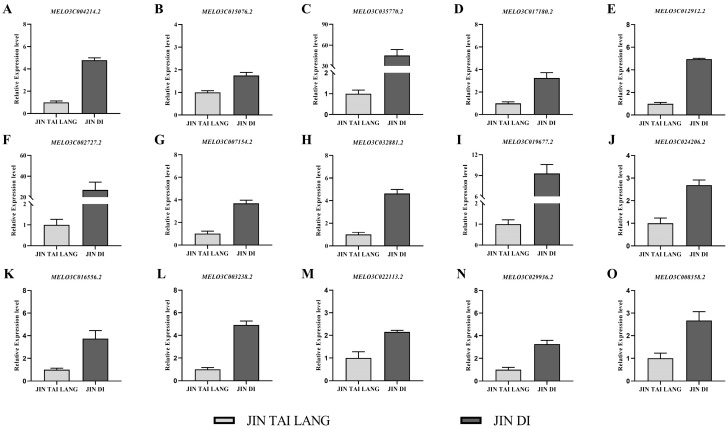
The quantitative real-time PCR (qRT-PCR) was used to analyze 15 DEGs in the most significant KEGG enrichment pathway in the ‘JIN TAI LANG’ and ‘JIN DI’ varieties. The corresponding gene expression levels of photosynthetic antenna protein are as follows: Lhca1 protein (**A**); Lhca3 protein (**B**); Lhca4 protein (**C**,**D**); Lhcb1 protein (**E**–**I**); Lhcb2 protein (**J**); Lhcb3 protein (**K**); Lhcb4 protein (**L**); Lhcb5 protein (**M**); Lhcb6 protein (**N**–**O**).Values are represented as the means ± SD (*n* = 9).

Through further analysis of KEGG enrichment pathways, we conducted fluorescence quantitative analysis on 15 different DEGs in the most significant “photosynthesis antenna proteins” enrichment pathway of the two varieties (Figure 8), and the results showed that 15 genes in the ‘JIN TAI LANG’ variety were significantly lower than those in the ‘JIN DI’ variety, and the expression trend was consistent with RNA-seq data (Figure 7D).

## 4. Discussion

In summer, the weather continues to be hot and rainy, and the crops often suffer from environmental stress of high temperature and high humidity. This stress often occurs in the seedling stage of melon, which causes serious harm to the growth of melon [41]. In this study, the HTH-sensitive ‘JIN TAI LANG’ and HTH-tolerant ‘JIN DI’ varieties were treated with HTH stress for 4 days, and it was found that the whole plant leaves of the sensitive melon ‘JIN TAI LANG’ variety gradually turned yellow and their growth was severely inhibited (Figure 1). However, the HTH-tolerant ‘JIN DI’ variety continued to maintain normal growth after 4 days of treatment but was less affected by HTH stress. These results indicate that two varieties of melon seedlings have different physiological responses to HTH stress, and different varieties have different adaptive adjustment mechanisms to resist HTH stress [42].

A large number of studies have shown that crops under stress can produce a large number of ROS, which can cause serious damage to the destruction of cell membranes and photosynthetic organs [43,44]. Plants all have antioxidant systems such as SOD, CAT, and POD that can rapidly remove reactive oxygen species to maintain the balance of reactive oxygen species in plants and reduce oxidative damage. In our study, under HTH stress, SOD, POD, APX, and CAT in the leaves of the two genotypes of melon all increased rapidly to remove excessive ROS produced (Figure 2). With the extension of stress time, CAT activity increased first and then decreased in the two varieties (Figure 2C), and APX decreased rapidly in the HTH-sensitive ‘JIN TAI LANG’ variety on the third day of stress (Figure 2D), indicating that the HTH-tolerant variety had stronger antioxidant capacity than the HTH-sensitive variety. Further analysis showed that there was no difference in the activity of the SOD enzyme between the two cultivars upon four days of stress (Figure 2A), while POD, CAT, and APX showed significant differences (Figure 2B–D), and it can be inferred that POD, CAT, and APX play an important role in the ‘JIN DI’ variety and alleviate the damage by increasing antioxidant capacity [45]. The content of MDA in the two genotypes of melon varieties decreased under HTH treatment (Figure 3A), which may be due to the increasing activity of antioxidant enzymes in leaves, leading to the decrease in MDA content of membrane peroxidation products, indicating that temporary extreme high temperature and high humidity cause little damage to membrane peroxidation. Chlorophyll content and REC value are important indexes to evaluate plant stress. In this study, we found that HTH stress led to a continuous decline in chlorophyll content and an increase in the REC value (Figure 3B,C), indicating that HTH stress inhibits the synthesis or degradation of chlorophyll while affecting the permeability of the cell membrane. The HTH-sensitive ‘JIN TAI LANG’ variety of chlorophyll contents and cell membrane damage is more serious. Those conclusions are consistent with previous results for wheat [46]. The soluble protein content in the two varieties increased first and then decreased (Figure 3D), indicating that melon synthesizes more protein to resist HTH stress at the beginning, but with the extension of stress, many proteins are degraded, and the resistance to high temperature and humidity decreases. When plants are subjected to high-temperature stress, the transpiration rate will accelerate the water dissipation of leaves, resulting in the death of leaves due to water shortages. In this study, it was found that the water content of leaves of the two varieties increased slightly under high temperature and high humidity (Figure 3E), which may be related to a higher relative humidity [47]. H_2_O_2_ and OFR have immune and signal transduction effects, but excessive accumulation will cause damage to the cell membrane and macromolecules. In this study, it was found that HTH stress caused a large amount of H_2_O_2_ and OFR accumulation in sensitive cultivars (Figure 3F,G), while the accumulation was not obvious in the HTH-tolerant ‘JIN DI’ variety with increasing antioxidant enzyme activity, indicating that HTH-tolerant cultivar had a better adaptive regulation mechanism.

Under high-temperature conditions, the most sensitive physiological process of plants is photosynthesis. When the temperature is higher than the optimum temperature, photosynthesis begins to decline gradually and reversibly, but at a certain critical temperature level, photosynthetic organs may be irreversibly damaged [48]. To further study the effects of HTH stress on photosynthesis, we measured the gas exchange parameters of the two melon varieties. Under HTH stress, the Pn of the HTH-sensitive ‘JIN TAI LANG’ variety decreased rapidly, and the photosynthetic capacity of the plants weakened. The cultivar with the HTH-tolerant ‘JIN DI’ variety maintained higher photosynthetic capacity, and the Pn decreased slightly (Figure 4A). Two melon seedlings of E, Gs, and Ci value were increased significantly (Figure 4B–D) and showed that melon under HTH stress conditions, by enlarging the stomatal conductivity, increased the concentration of carbon dioxide in the cell, improved the photosynthetic rate, transpiration rate and speed up to reduce the temperature of the blade, and mitigated the effects of HTH stress, and this defense of plants against high temperature is similar to that found in previous studies of wheat [49] and tomatoes [50].

Photosystem II (PSII) is considered to be one of the most sensitive components of photosynthetic organs [51]. In this study, we found that the Fv/Fm, qP, Y(II), and ETR(II) indexes of HTH-sensitive ‘JIN TAI LANG’ variety decreased under HTH stress and decreased rapidly with the extension of stress days (Figure 5). The results showed that under the HTH stress, the photosynthetic activity of the photosystem II reaction center in the open state of the ‘JIN TAI LANG’ variety was lower, the light energy efficiency captured by the open PSII reaction center was lower, the maximum light energy conversion efficiency and actual light energy conversion efficiency were significantly affected, and the relative electron transfer rate of photosystem II was slower, which was consistent with the conclusion of previous studies [52]. The NPQ index of the two cultivars also decreased gradually under stress, indicating that the extremely HTHstress resulted in the destruction of the photosynthetic structure, reduced the photoprotective ability to dissipate excess light energy as heat, and gradually lost the ability of heat dissipation [53]. The photochemical efficiency Y(I) and relative electron transfer rate ETR(I) of PSI in the two melon varieties also decreased rapidly, while Y(ND) and Y(NA) increased, indicating that the excitation energy was transferred from PSII to PSI and converted to the photochemical quantum yield Y(I) decreased under HTH stress. The non-photochemical energy Y(ND) and Y(NA) that dissipated increased, and with the extension of stress time, Y(NA) decreased first and then increased, which may lead to damage to the CO_2_ fixation level and enhance the receptor side limitation. Chloroplasts are the main reaction sites of photosynthesis, and the photosynthetic intensity of leaves is closely related to the ultrastructure of chloroplasts. By analyzing the ultrastructure of chloroplasts in the leaves of the two cultivars on day 3 of stress (Figure 6), we found that the cells and chloroplasts of the sensitive cultivars were seriously damaged, the inner capsule tissue was damaged, the accumulation of starch granules increased, and the number of plastid pellets increased [54]. The results showed that combined high temperature and high-humidity stress damaged the integrity of the chloroplast, severely damaged the PSI and PSII systems, and inhibited the light energy absorption, conversion, and electron transfer efficiency, which was similar to that in previous results [55,56]. However, the HTH-tolerant ‘JIN DI’ variety was only slightly damaged, and the chlorophyll fluorescence index was slightly decreased, which further explained the difference between the sensitive cultivars and the HTH-tolerant cultivars in photosynthetic organelles under HTH stress, which was worthy of further study.

To further explain the difference between the HTH-sensitive ‘JIN TAI LANG’ variety and the HTH-tolerant ‘JIN DI’ variety under HTH stress, transcriptome sequencing was performed on the two cultivars under stress for three days, and a total of 3390 DEGs were obtained. A total of 1647 up-regulated genes and 1744 down-regulated genes were used as references for the ‘JIN TAI LANG’ variety. Through GO functional enrichment analysis and KEGG pathway enrichment analysis, it was found that the 15-genes in the pathway with the most significant differences between varieties were down-regulated in ‘JIN TAI LANG’ compared with ‘JIN DI’ (Figure 7D). According to the KEGG pathway diagram, these 15 genes are key genes in the synthesis of a light-harvesting chlorophyll protein complex (LHC) (Figure 7E), and the LHC protein is mainly involved in light harvest and light protection [57]. The Lhca1, Lhca3, and Lhca4 proteins in photosystem II and Lhcb1-Lhcb6 protein synthesis related genes in photosystem I were inhibited in the HTH-sensitive ‘JIN TAI LANG’ variety compared with those in HTH-tolerant ‘JIN DI’ variety. Therefore, it was concluded that the decreased decomposition of antenna protein content was greater than synthesis under HTH stress in the ‘JIN TAI LANG’ variety, and the light energy capture ability was decreased, and the light energy captured by photosystems I and II was insufficient to supply the consumption of ATP and NADPH required by normal plants [58], leading to a decrease in photosynthesis, photosynthetic efficiency and electron transfer efficiency. In addition, the degradation of LHC antenna proteins also impaired the heat dissipation function of photosynthetic organs in the ‘JIN TAI LANG’ variety under HTH stress, and excess heat in the photosystem could not be dissipated, destroying the photodamage defense mechanism and resulting in more damage to the HTH-sensitive variety than to the HTH-tolerant variety [59].

## 5. Conclusions

Under HTH stress, SOD, POD, and APX activities of two different genotypes of melon seedlings increased rapidly to remove excess ROS to cope with the damage caused by HTH stress, and the HTH-tolerant ‘JIN DI’ variety had a stronger antioxidant capacity. With the extension of stress time, chlorophyll content and soluble protein in leaves of the HTH-sensitive ‘JIN TAI LANG’ variety decreased rapidly, the cell membrane permeability increased, and the H_2_O_2_ and OFR content increased compared with the HTH-tolerant ‘JIN DI’ variety, indicating that the stress was more serious. The Pn, Fv/Fm, qP, Y(II), ETR(II), Y(I), and ETR(I) of the ‘JIN TAI LANG’ variety were significantly inhibited and decreased with the extension of stress time, while the indexes of the tolerant cultivars decreased slightly and maintained a high value, and showed a better photosynthetic coping mechanism to adapt to HTH stress. By comparing chloroplasts of different varieties under three days of stress, it was found that chloroplasts of sensitive varieties were significantly degraded, starch grains and osmophilic grains were increased, and matrix and grana were seriously damaged, while chloroplasts of tolerant varieties were degraded only a few times and were slightly stressed. Transcriptional analysis showed that the Lhca1, Lhca3, and Lhca4 proteins in photosystem II and the Lhcb1-Lhcb6 protein synthesis genes in photosystem I were inhibited, resulting in decreased light energy capture ability and more severe damage to the heat dissipation mechanism in the ‘JIN TAI LANG’ variety. The damage degree of chloroplasts and the degradation degree of capturing antenna proteins in different genotypes under HTH stress may be the main reason for the decrease in photosynthesis parameters and the severe damage to chloroplasts in different genotypes of melon. This study also provides a basis for future studies on the HTH stress tolerance mechanism of melons and provides new insights for the HTH-tolerant breeding of melons.

## Data Availability

The RNA sequence data set are available in the repository of NCBI Sequence Read Archive (SRA) under the GenBank accession BioProject: PRJNA775054 and accession number: SRR16596901-16596906.

## Supplementary Materials

The following are available online at https://www.mdpi.com/article/10.3390/ijms23020734/s1.

## Abbreviations

HTH: high temperature and humidity; Pn: net photosynthetic rate; E: transpiration rate; Gs: stomatal conductance; Ci: intracellular CO2 concentration; SOD: superoxide dismutase; POD: peroxidase; CAT: catalase; APX: ascorbate peroxidase; MDA: malondialdehyde; ROS: reactive oxygen species; OFR: superoxide anion.
