# Supplementary material for: Physiological and Transcriptomic Analysis Reveals the Responses and Difference to High Temperature and Humidity Stress in Two Melon Genotypes
Source: Int J Mol Sci. 2022 Jan 10;23(2):734. doi: 10.3390/ijms23020734 (PMC8776189; doi:10.3390/ijms23020734)
Supplement: Supplementary file 1 [file ijms-23-00734-s001.zip › Table S1 List of primers used for qRT-PCR.pdf]

Table S1. List of primers used for qRT-PCR.

Gene code	Forward Primer	Reverse Primer	Gene description
MELO3C004214.2	GGCTTCCAACACCTTGAT	GGTTCTCTGGCACTTCTC	*Lhca1*
MELO3C015076.2	TGCTGCCAGACAGAGATT	GATCACTTCGCCGTATGC	*Lhca3*
MELO3C035770.2	CTTCCAGCACAGTCTTCC	GCGTGTACCACCTAAGTT	*Lhca4*
MELO3C017180.2	CGTCAACTCTGTTCGTAATC	CATTGGCTAGTTCCTTCTCT	*Lhca4*
MELO3C012912.2	GTGAGCCTCCATCTTACC	TTGTGAACCAGCCTTGAA	*Lhcb1*
MELO3C002727.2	TCGGAGAAGCAGTATGGT	GCAAGATGGTCAGCAAGA	*Lhcb1*
MELO3C007154.2	TCCACCACCTCCAACAAT	CACCGAACTTCACACCAT	*Lhcb1*
MELO3C032881.2	GAGCCACCATCCTACCTT	CCATTCTTCAACTCCTTAACC	*Lhcb1*
MELO3C019677.2	GCCAAGCCGTCAAGTTAT	CAATTCTGTATCCTTCCACTG	*Lhcb1*
MELO3C024206.2	ACGCCATCTTACCTGACT	ATTGCCAGCCTTCCATTC	*Lhcb2*
MELO3C016556.2	GTATGGTTCAAGGCAGGAG	CAAGGTGGTCAAGAAGGTT	*Lhcb3*
MELO3C003238.2	GCAACCTCCTCCTTCATC	GTGAGCCATTCAACAGAGA	*Lhcb4*
MELO3C022113.2	CTCAGCAAGAAGCCAGAA	GCCACCAACAAGAACAAC	*Lhcb5*
MELO3C029936.2	ATTCCACTCCAGGCTTGT	CTTGTTCCATTCGTAGACTTC	*Lhcb6*
MELO3C008358.2	CCGTGGAAGTAGCAGAAC	CGAAGGAGAATGGAGCAAT	*Lhcb6*
Actin	TCTATTCCAGCCATCTCTC	GACCCTCCAATCCAAAC	
